# Precisional detection of lymph node metastasis using tFCM in colorectal cancer

**DOI:** 10.1515/biol-2022-0780

**Published:** 2023-12-13

**Authors:** Dan Yang, Jingling Tang, Yunhuan Zhen, Jindong Yuan, Pingsheng Hu, Xiaoyun Li, Hang Du, Xiaolan Zhang, Yuan Yang

**Affiliations:** Clinical Medical Research Center, The Affiliated Hospital of Guizhou Medical University, No. 28 Guiyi Road, Yunyan Zone, Guiyang 550004, Guizhou, China; Department of Anorectal Surgery, The Affiliated Hospital of Guizhou Medical University, Guiyang, Guizhou, China; Department of Research and Development, Sinorda Biotechnology Co., Ltd, Guiyang, Guizhou, China

**Keywords:** flow cytometry, lymph node, metastasis, colorectal cancer

## Abstract

The detection of colorectal cancer (CRC) lymph node (LN) metastases significantly influences treatment choices, yet identifying them in samples is time-consuming and error-prone. To enhance efficiency, we have established a LN metastasis detection method utilizing triple-parameter flow cytometry (tFCM) and have conducted a comparative assessment of its accuracy and cost-effectiveness in contrast to conventional pathological examinations. This technique utilized biomarkers cytokeratin 20 (CK20), epithelial cell adhesion molecules (EpCAM), and Pan-CK. tFCM’s sensitivity was validated by analyzing known cell line concentrations (SW480 and SW620) in peripheral blood mononuclear cells (PBMCs), with CK20, EpCAM, and Pan-CK showing significant expression in CRC cell lines but not in PBMCs. A strong linear correlation was observed in the mixed leukocyte environment (*R* ^2^ = 0.9988). Subsequently, tFCM and pathological sections were employed to analyze LNs from CRC patients, enabling comparison of detection accuracy. Within the 36 LNs studied, tFCM successfully identified tumor cells with varying metastasis degrees, including micro-metastasis and isolated tumor cell clusters. Notably, relying solely on pathological sections led to a potential 25% misdiagnosis rate for LNs. In contrast, tFCM effectively minimized this risk. In summary, compared to traditional pathological sections, tFCM is a more advantageous method for detecting nodal metastasis in CRC patients, offering a more precise prognosis for these patients.

## Graphical abstract

**Figure:**
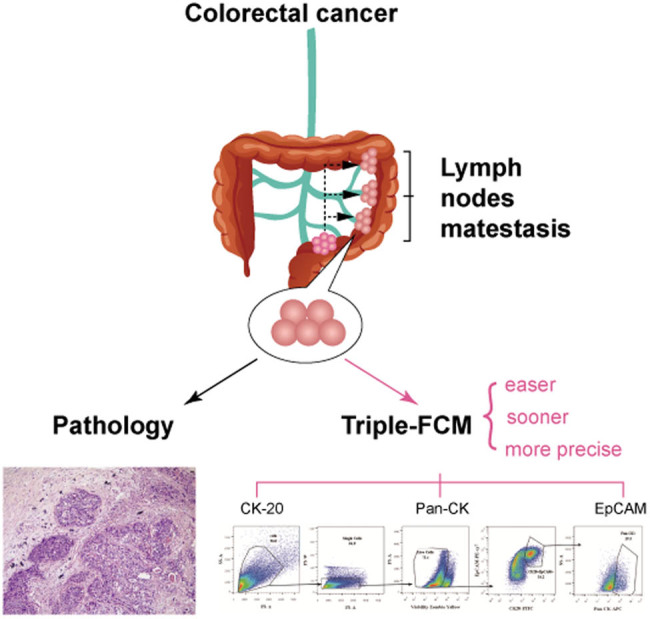

## 1 Introduction

Colorectal cancer (CRC) stands as the predominant malignancy affecting the digestive system globally, with a staggering 1.9 million new cases and 935,000 fatalities recorded worldwide in 2020 [1]. Notably, lymph node (LN) metastases hold paramount significance in terms of treatment response, survival rates, and unfavorable prognostic indicators among CRC patients. A previous investigation unveiled that 32.9% of individuals with CRC exhibited LN involvement leading to local recurrence [2]. Although conventional pathological sections serve as the established means of detecting LN metastases for nodal staging, their accuracy heavily relies on the expertise of pathologists and subjective assessments made under the microscope. Furthermore, they suffer from an inherent limitation, namely the inability to examine larger LNs [3]. Moreover, micro-metastasis (MIC) and isolated tumor cell clusters (ITCs) constitute unfavorable prognostic factors [4,5] and are considered crucial in nodal staging [6]. Unfortunately, the identification of MIC and ITC often eludes conventional pathological sections, even when employing a single marker, such as immunohistochemistry (IHC) [7]. Given the aforementioned challenges, there arises a need for a more rapid and accurate approach to staging that can provide clinical feedback to guide post-surgical treatments for CRC patients.

In recent years, novel techniques such as quantitative real-time polymerase chain reaction (qRT-PCR) [8,9] and the one-step nucleic acid amplification (OSNA^®^) assay have emerged for detecting metastases in sentinel nodes (SLNs), surpassing the sensitivity of pathological sections [10]. Nevertheless, both RT-PCR and OSNA^®^ exhibit drawbacks, including their reliance on minimal tissue samples, susceptibility to selection bias, and high costs [11]. In contrast, flow cytometry (FCM) presents a simpler, more comprehensive, and time-saving method for sample processing. Studies led by Ola.Winqvist utilized tumor molecular markers in single-parameter or combined double-parameter FCM to detect tumor cells infiltrating LNs in renal cancer [12] and penile cancer [13]. Despite showing promise in nodal staging, FCM still necessitates refinement for the precise detection of varying degrees of LN metastases [14]. Studies have indicated that the expression of Pan-CK in tumor cells correlates with distant metastasis, lymphatic invasion, and tumor budding [15]. Cytokeratin 20 (CK20), on the other hand, serves as the most prevalent immunohistochemical molecular marker employed for routine cancer staging in clinical pathology laboratories [16]. Epithelial cell adhesion molecules (EpCAM), specifically highly expressed in CRC, exhibit negligible expression in both normal and malignant epithelia. As a diagnostic marker, EpCAM has found utility in detecting mature carcinoma cells in mesenchymal organs like blood, bone marrow, or LNs, and particularly in assessing rare circulating tumor cells in carcinoma patients [17].

LN represents immune organs composed of diverse cell populations, while normal gastrointestinal tissues exhibit varying degrees of tumor marker expression. Consequently, employing multiple markers concurrently becomes imperative to accurately identify tumor cells within LNs and minimize staining background originating from immune cells.

In this study, we sought to combine three tumor markers, CK20, EpCAM, and Pan-CK, through triple-parameter FCM (tFCM), with the aim of evaluating whether tFCM could enhance the precision and efficiency of LN metastasis detection in CRC patients.

## 2 Materials and methods

### 2.1 Patients

Twenty-two CRC patients (14 men, 8 women; mean age, 60.95 ± 11.36) enrolled between November 2017 and January 2019 and scheduled for primary surgical treatment were included in this study. The characteristics of the included CRC patients are reflected in Table 1, where “nodes in PAD” represent metastasis/total number of LNs in pathological anatomical diagnosis from LN dissection [18]. Inclusion criteria: patients diagnosed with colon or rectal cancer and requiring surgical resection for treatment.

**Table 1:** Characteristics of patients with CRC

Case number	Age	Gender	Diagnosis	T stage	N stage	Dissection nodes in PAD	Tumor Size (cm)
1	38	Male	Rectal cancer	T3	N2b	11/24	5 × 5
2	64	Female	Colon cancer	T4b	N2a	/	5 × 4
3	65	Female	Rectal cancer	T3	N2a	4/12	4 × 3
4	53	Female	Colon cancer	T4b	N2b	16/37	10 × 8
5	77	Male	Colon cancer	T3	N2a	4/12	7 × 6
6	43	Male	Colon cancer	T3	N0	0/11	4.0 × 3.0
7	52	Male	Rectal cancer	T3	N0	/	8 × 6
8	51	Female	Rectal cancer	T3	N0	0/12	4 × 3
9	71	Male	Rectal cancer	T2	N0	0/9	1.5 × 4
10	74	Female	Rectal cancer	T2	N0	0/10	8 × 1.4
11	52	Male	Rectal cancer	T3	N0	0/12	2 × 1.8
12	72	Male	Rectal cancer	T2	N0	0/10	3 × 3
13	62	Female	Rectal cancer	T3	N1a	1/13	3 × 2 × 1
14	66	Male	Rectal cancer	T3	N0	0/17	2 × 3; 4 × 2
15	58	Male	Rectal cancer	T3	N0	0/10	4 × 3
16	53	Male	Colon cancer	T3	N2a	4/24	5 × 7
17	53	Male	Colon cancer	T3	N1b	3/24	6 × 5 × 5
18	76	Male	Rectal cancer	T4b	N2b	/	4 × 4
19	75	Male	Rectal cancer	T3	N1	2/8	5 × 6
20	73	Male	Colon cancer	T3	N0	0/10	3 × 3 × 1.5
21	46	Female	Rectal cancer	T3	N2b	8/19	3 × 2.8 × 0.6
22	67	Female	Colon cancer	T3	N0	0/11	3 × 2 × 0.6

N staging of CRC: N0: There is no regional LN is tumor cell metastasized; N1: 1–3 regional LNs are metastases; N1a: 1 regional LN is metastasis; N1b: 2–3 regional LNs are metastases; N2a: 4–6 regional LNs are metastases; N2b: 7 and more LNs are metastases. Nodes in PAD: metastasis/total number of LNs in pathological anatomical diagnosis from LN dissection.

### 2.2 Cell line

Two colorectal adenocarcinoma cell lines were used in this study. SW480 and SW620 (ATCC, USA) were cultured in L15 medium (Gibco, USA) at 37°C in a humidified atmosphere of 95% air with 5% CO_2_. The medium was supplemented with 10% fetal bovine serum (BGS, Gibco, USA) and 1% penicillin/streptomycin (Hyclone, USA). SW480 was established from a primary adenocarcinoma of the colon. SW620 was derived from a LN in the same manner as SW480.

### 2.3 Negative control samples and peripheral blood mononuclear cells (PBMCs)

Three tumor-cell-free LNs from appendicitis patients after appendectomy were set as negative control samples. PBMCs were obtained from the venous blood of medically examined healthy volunteers without tumors and infectious diseases and were isolated using the Ficoll-Paque gradient method by Ficoll (Solarbio, China).

### 2.4 LN acquisition

LNs were obtained from the patients after colonic surgery; 1 mL of methylene blue was injected subserosal into four areas around the tumor. A few minutes later, the first blue LN is SLN, and the subsequent colored one is non-SLN. Each LN was cut in half through the largest diameter. Half of the LN was placed in DMEM medium supplemented with 10% BGS immediately, cut into pieces, and digested by enzymatic dissociation to obtain single-cell suspensions that could be analyzed by FCM. The other half was soaked in 10% paraformaldehyde diluted by double-distilled water for 8 h to prepare pathological sections in parallel.

### 2.5 Identification of CRC cells in mixed cultures

To simulate the environmental condition of metastatic LNs, tumor cells SW480, established from a primary adenocarcinoma of the colon, were added to a PBMCs suspension in a three-fold stepwise dilution series (0.9, 0.3, 0.1, 0.0333, 0.0111, 0.0037, and 0.0012%). PBMCs alone served as a negative control. The corresponding markers were then measured using the FCM assay.

### 2.6 FCM

Single-cell suspensions were resuspended in zombie yellow live/dead dye diluted 1:500 in phosphate buffer saline to mark dead cells. Fc inhibitor blocked the unwanted staining of FcR on the cells. After blocking, the antibodies were added for EpCAM surface staining [configured PE-cy7 (excitation laser 488 nm, Emission wavelength 767 nm), Biolegend, USA] and incubated for 30 min. The stained cells were fixed and permeabilized with the Cytofix/Cytoperm kit (BD, USA). Antibody for Pan-CK intracellular staining [configured APC (Excitation laser 640 nm, Emission wavelength 660 nm), Abcam, UK] and CK20 [configured FITC (Excitation laser 488 nm, Emission wavelength 520 nm), Abcam, UK] were incubated for 30 min. The cells were acquired by FCM with the help of the Beckman Navios (Beckman coulter, USA) using the software FlowJo (TreeStar, Ashland, OR).

### 2.7 Pathology

The dehydrated LNs were embedded in paraffin following pathologic routine methods. Every paraffin block was cut into 4-μm sections and adhered to a glass slide. The paraffin sections were deparaffinated in xylene and successively rehydrated in alcohol. All the sections were stained for H&E first. If the metastases in the H&E staining sections were negative, IHC was performed on the unstained consecutive sections with a Pan-CK antibody. 10 mM citrate buffer was used as a heat-mediated antigen retrieval and blocked with 10% goat serum for 30 min at room temperature, followed by incubation with Pan-CK (diluted 1/400, Abcam, UK) at 4°C overnight. Anti-mouse horseradish peroxidase polymer was used as the secondary detection system. The LN H&E and IHC results were photographed. The size of the tumor cell cluster was measured by Adobe Photoshop software.

### 2.8 Clinical nodal staging

Metastases of H&E-stained and IHC-stained LN slides were reviewed independently according to the 8th edition of the American Joint Committee on Cancer staging manual. Tumor cell clusters with a maximum size greater than 2.0 mm are considered macrometastasis (MAC), larger than 0.2 mm but not larger than 2.0 mm or 10–20 tumor cells in the cluster are MIC, smaller than 0.2 mm or single cells are ITC [19].

### 2.9 Statistical analysis

Each experiment was performed at least three times. Graphpad prism7 and SPSS were applied for analyzing data. The correlation between detected and added CRC cancer cell line was calculated by linear regression of Prism. The value was presented as \[\bar{X}]\] ± SD.

The data tested by SPSS did not conform to the normal distribution, and the variance was not uniform. Therefore, to compare the percentage of positive cells in each group, the Kruskal Wallis of the non-parametric test was chosen to derive the *P* value. *P* < 0.05 was considered a statistical significance. Data were evaluated by SPSS for Kappa, sensitivity, specificity, and likelihood ratio of the tFCM method. The relationship between patients’ clinical factors and tFCM diagnosis was assessed by Fisher’s exact test of SPSS.


**Informed consent:** Informed consent has been obtained from all individuals included in this study.
**Ethical approval:** The research related to human use has been complied with all the relevant national regulations, institutional policies and in accordance with the tenets of the Helsinki Declaration, and has been approved by the Ethics Committee of the Affiliated Hospital of Guizhou Medical University, (approval No. 2014-40, from 7 March 2014).

## 3 Result

### 3.1 Detection of tumor cell markers of CRC

**Figure 1:**
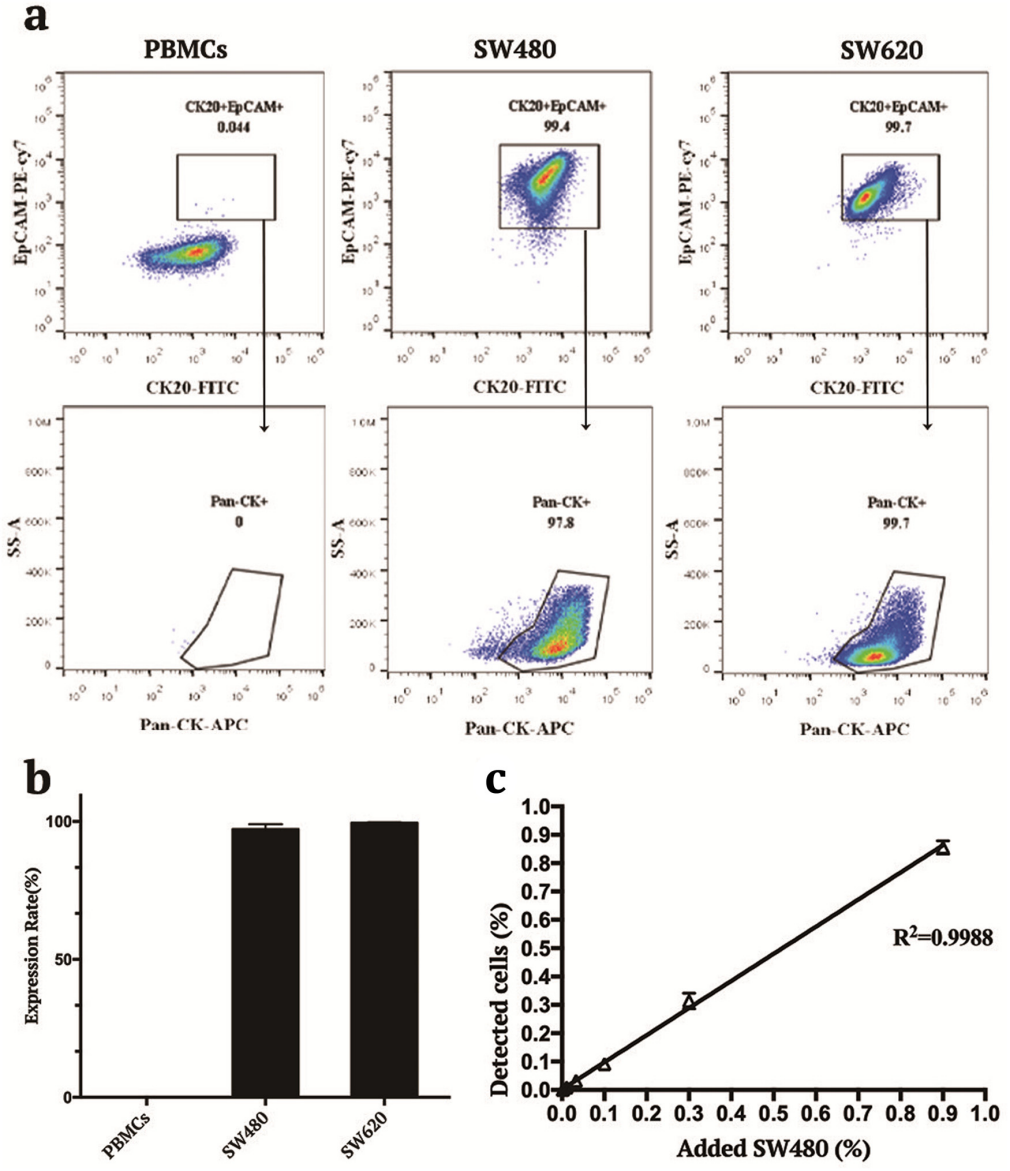
The capacity of tFCM in detecting positive events in CRC cell levels. (a) Two CRC cell lines SW480 and SW620 were stained by tumor marker antibodies CK20, EpCAM, and Pan-CK. PBMCs were used as background staining. FCM detected tumor markers’ positive events in cells. With the remaining Pan-CK and EpCAM common positive events, CK20-positive events were gated, which were CK20-EpCAM-PanCK-positive events shown in different cell lines. (b) Statistics of CK20-EpCAM-PanCK-positive events in CRC cell lines and PBMCs. (c) PBMCs suspension was spiked with the tumor cells SW480 with three-fold stepwise dilution series. *R* ^2^ showed the correlation between added SW480 and detected CK20-EpCAM-PanCK-positive events to analyze the accuracy and sensitivity of three tumor markers by FCM in a mixed leukocyte environment. A representative experiment with \[\bar{X}]\] ± SD of 3 replicates is presented.

The expression of CK20, Epcam, and Pan-CK in two different CRC cell lines, SW480 and SW620, was investigated by FCM. PBMCs were derived from healthy people’s blood set as background cells. The combination of three tumor markers (CK20-Epcam-Pan-CK) was expressed at >97% in two tumor cell lines, but there were no positive events at all (0%) in PBMCs (Figure 1a and b).

### 3.2 Detection of CRC cells in a mixed leukocyte environment

To simulate the environmental condition of metastatic LNs, PBMCs suspension and the tumor cells SW480 were diluted in the three-fold stepwise dilution series (0.9, 0.3, 0.1, 0.0333, 0.0111, 0.0037, and 0.0012%). The tFCM method showed good stability with the coefficient of variance (CV) ranging from 2.75 to 20.91% when detecting the high concentration of SW480 (0.9–0.1%). As the cell concentration decreased, the CV value gradually increased (data not shown). Furthermore, assuming an LN size of approximately 10 × 5 × 5 mm, an MIC of 2 mm is equal to 1.6% of the total number of cells in the average LN. Thus, an MIC of 0.2 mm is equivalent to 0.032% of the total number of cells [13]. The regression line of CK20-EpCAM-Pan-CK showed high accuracy (Figure 1c) in terms of MIC and ITCs concentrations (0.1–0.0012%) and no unspecific background staining, indicating that the combination of the three tumor makers in FCM is reliable and sensitive for detecting metastatic tumor cells at different concentrations in LNs.

### 3.3 Gating strategy for tFCM-positive tumor cells

**Figure 2:**
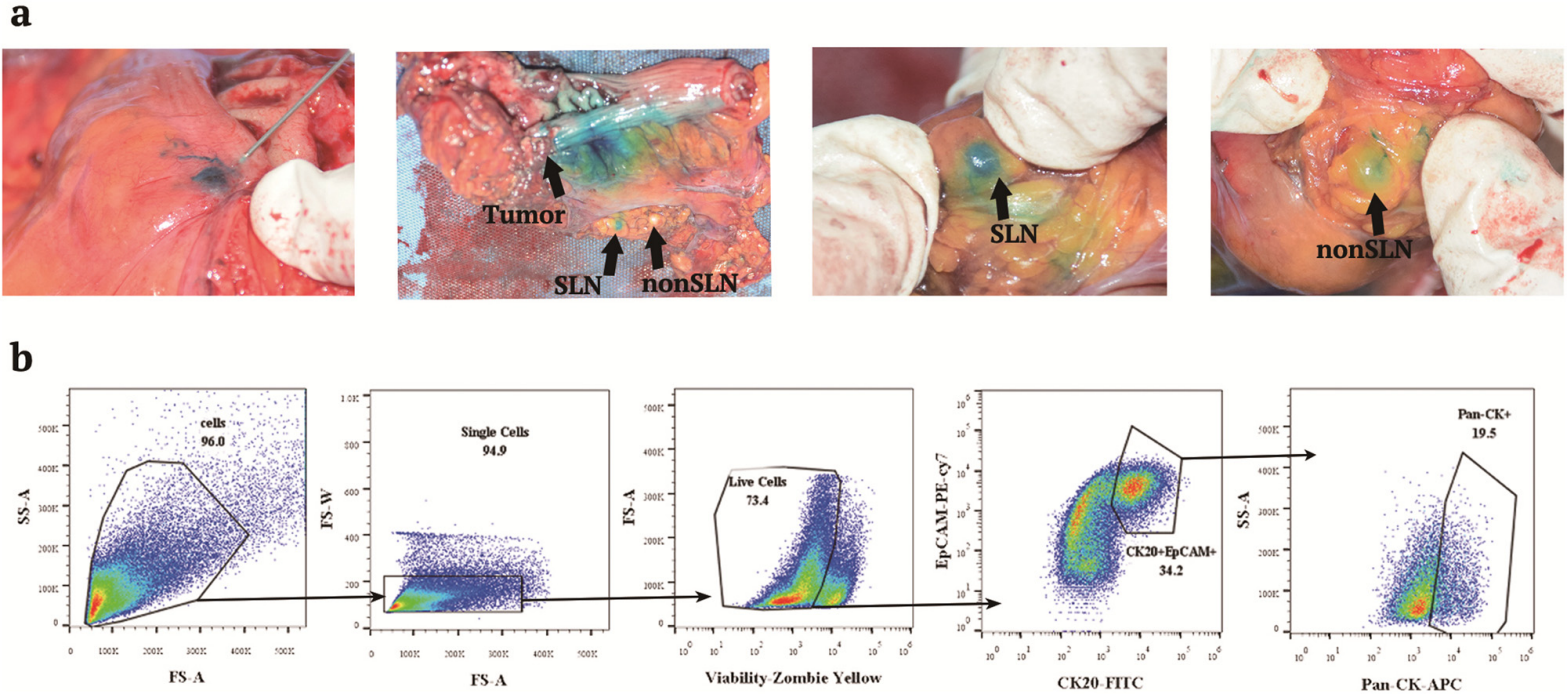
Identification of SLN from CRC patients and FCM gating strategy of tumor cells in LN. (a) During colorectal surgery, patent blue dye was injected around the primary tumor within about 5 min. The first blue-drained LN was SLN, and the next blue LN was non-SLN. (b) The gating strategy of tumor cells in LN: In the first step, cell debris is gated off by FS-A versus SS-A axis. Single cells are gated in from cells in the second. Live cells are gradually gated depending on viability-FMO control (viability-fluorescence minus One, sample stained with all antibodies except viability zombie yellow). From the live cell gate, double molecular positive cells were gated on a CK20 versus EpCAM plot based on the CK20-FMO control and EpCAM-FMO control gating location, respectively. From this double molecular positive population, the frequency of Pan-CK-positive cells was quantified by Pan-CK-FMO control, and the CK20-EpCAM-Pan-CK triple-positive cells represent the tumor cells among the whole population of cells.

Positive events were gated depending on fluorescence minus one control. The gating strategy of triple molecular marker FCM as a sample is shown in Figure 2b. CK20-EpCAM-Pan-CK-positive events were noted as tFCM-positive tumor cells (Figure 2b).

### 3.4 FCM and pathology detection of metastatic cells in SLN

**Figure 3:**
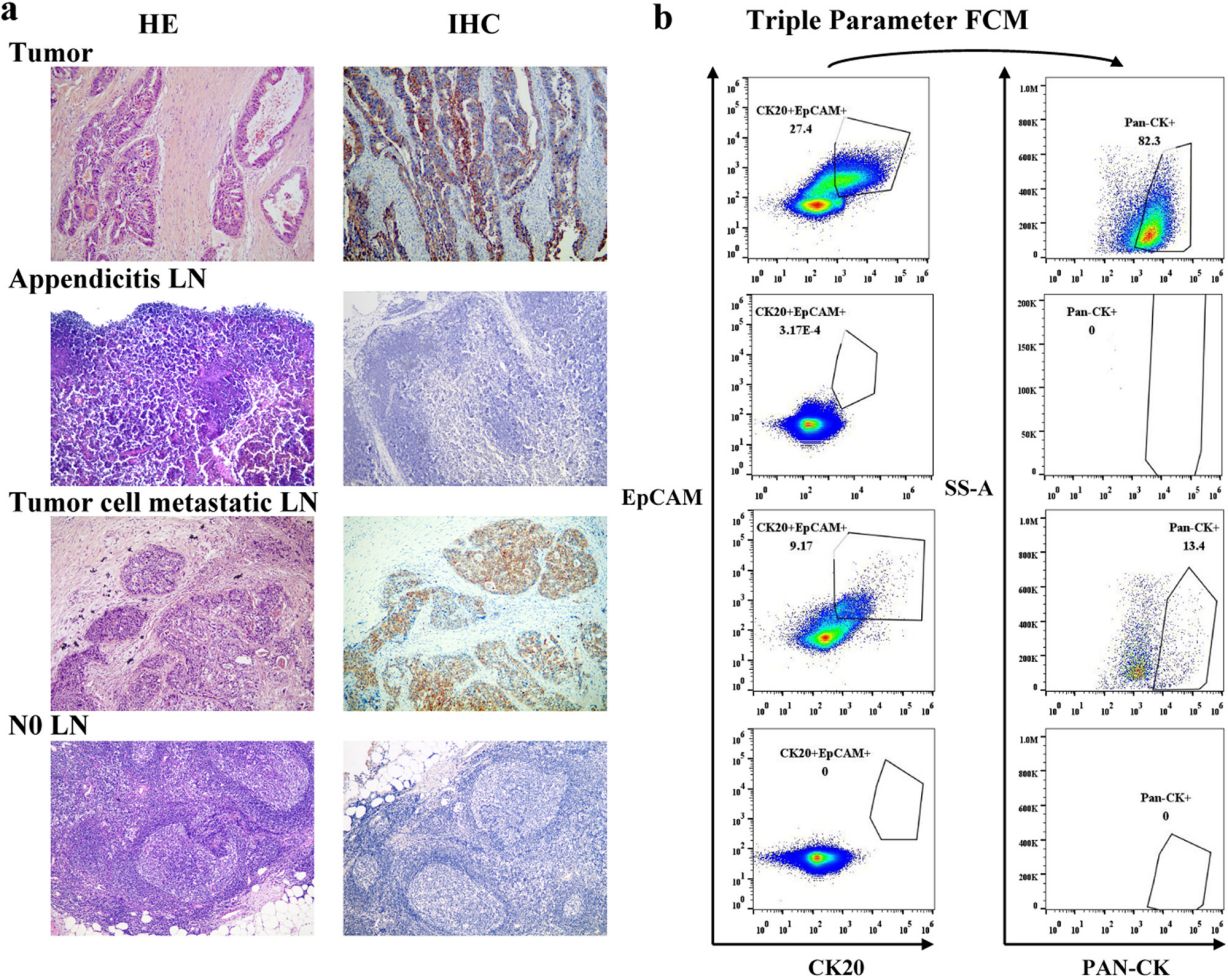
Metastasis in LNs detected by pathologic section and FCM. The tumor was used as the positive control. LNs from appendicitis patients were set as negative controls. (a) Sections of LNs stained with H&E combined with IHC were examined by the microscope (×100). (b) The gating strategy applied to FACS data analysis: First, CK20 and EpCAM-positive cells were gated, and from this subpopulation, the frequency of Pan-CK-positive cells was quantified.

Using tumor tissue as the positive control and appendicitis LN as the negative control, we have conducted a comparative analysis of LN metastasis through H&E staining, IHC (Figure 3a), and tFCM techniques (Figure 3b). The tumor cell cluster in MAC LNs could be observed under the microscope in one H&E-stained section (Figure 3a). As depicted in the illustration, the positive outcomes of H&E and IHC in tumor tissue and LNs with tumor metastasis are recapitulated in tFCM, whereas the negative results of appendicitis LNs and N0 LNs are replicated in tFCM. FCM detected positive events in the four MAC LNs simultaneously (Table 2). After a series of continuous pathological sections, three MIC LNs and three ITC LNs were observed by a microscope in the IHC sections (Table 2). No tumor cells were observed in 17 LNs either by H&E or IHC and nor did FCM detect any positive events in these pathologically negative LNs (Figure 3b, N0 LN). There were nine pathologically negative LNs and FCM detected positive events in the LNs (Table 3). The positive events of the nine pathologically negative LNs did not differ in ITC LNs and MIC LNs (*Kruskal Wallis-test, P = 1 and P = 1*). The nine pathologically negative LNs tended to be metastatic LNs. Among the 22 patients, the nodal staging was updated to metastasis in 7. Two of the seven patients died 3 years after CRC resection (Table 3). The statistics of LN metastases between triple marker FCM and pathology are given in Table 4. Nine of the 36 LNs were misdiagnosed as negative by pathological sections, meaning that 25% of LNs had a risk of being misdiagnosed by pathological sections. The statistics indicated that FCM could detect tumor cells with varying degrees of metastases to LNs in CRC patients, including MIC and ITC LNs and the metastases that could not be detected by pathology.

**Table 2:** The comparison between different metastatic degree LNs examined by pathology and FCM

Case number	Samples	Largest dimension of tumor cell cluster(mm)	Number of tumor cells draining in LN	The type of metastasis	Positive events detected by FCM (%)
1	SLN	>2	/	MAC	1.3000
2	SLN	>2	/	MAC	0.7200
3	NonSLN	>2	/	MAC	0.0850
4	NonSLN	>2	/	MAC	0.0519
5	SLN1	0.0371	17	MIC	0.0720
6	SLN	0.0170	4	ITC	0.0008
	NonSLN	0.1022	56	MIC	0.0242
7	SLN	0.0840	12	MIC	0.0094
8	SLN	0.0223	6	ITC	0.0004
9	SLN	0.0474	8	ITC	0.0005

**Table 3:** Nodal staging and prognosis in positive detected by 3pFCM but negative by pathology LNs

Case number	Samples	FCM	Pathology	FCM nodal staging	Pathology nodal staging	Prognosis
5	SLN1	+	+	N1	N1a	Recover
	SLN2	+	−			
10	NonSLN	+	−	N1a	N0	Died
15	SLN1	+	−	N1	N0	Recover
	NonSLN	+	−			
16	SLN	+	−	N1a	N0	Died
17	SLN	+	−	N1	N0	Recover
	NonSLN1	−	−			
	NonSLN2	−	−			
	NonSLN3	+	−			
20	NonSLN	+	−	N1a	N0	Recover
22	NonSLN	+	−	N1a	N0	Recover

+: Tumor cells detected in LN; −: No tumor cells detected in LN.

**Table 4:** Statistics between triple marker FCM and pathology

		Pathology		Total
		Positive	Negative	
FCM	Positive	10	9	19
	Negative	0	17	17
Total		10	26	36

**Figure 4:**
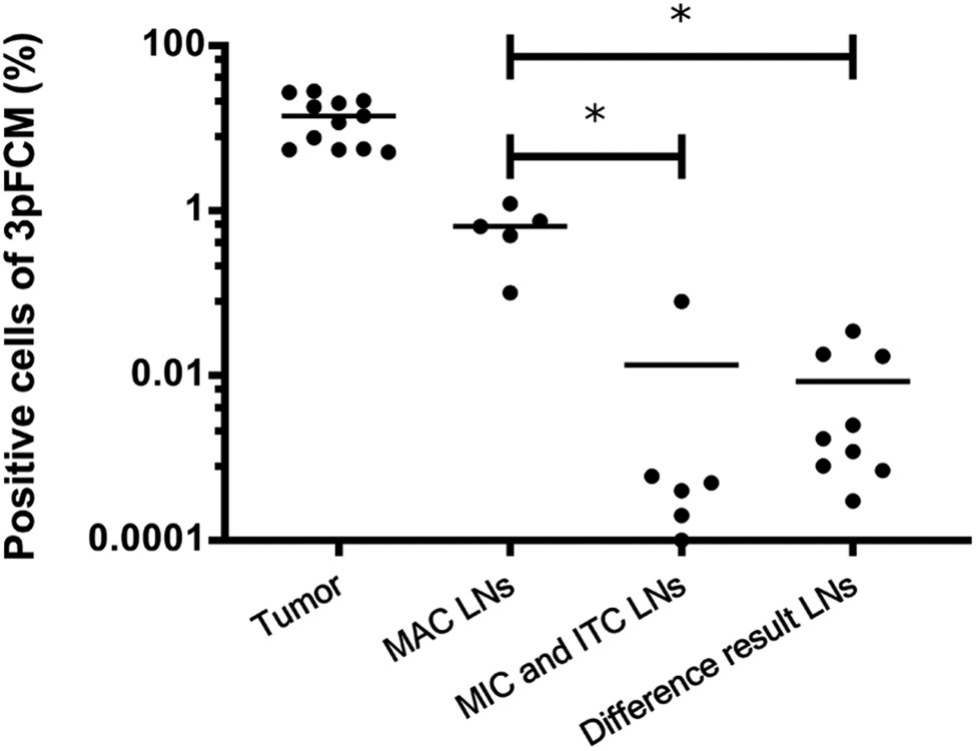
Summary of LN triple marker FCM results. The frequency of FCM-positive cells in MAC LN, MIC, and ITC LN, and results of differences in LNs between FCM and pathologic section. These samples were analyzed by SPSS software. Kruskal Wallis-test, *P < 0.05.

Pathology was designated as the criterion for detecting LN metastases in statistics. SPSS statistical analysis showed moderate agreement between FCM and pathological sections (*Kappa = 0.512*). The sensitivity and specificity of FCM were 100 and 65.38%, respectively. The positive and negative likelihood ratios of FCM were 2.889 and 0. From the earlier analysis, it could be concluded that there was no risk of missed detection and no false negatives for tFCM (Figure 4).

### 3.5 Correlation analysis of LN metastases detected by FCM with clinicopathology

The distribution of the clinicopathological factors between patients with pathologically detected tumor cell-metastatic nodes and those without tumor cell nodes was analyzed to define the correlations with LN metastases. The results revealed that LN metastases detected by FCM were significantly associated with cancer type (*Fisher’s exact test, P = 0.022*). Colon cancer patients were more likely to have metastases in their LNs than rectal cancer patients. Colon cancer is more likely to present with lymph vascular invasion than rectal cancer. These findings could not be observed by pathological examination (*Fisher’s exact test, P = 0.662*). In addition, the tumor size of node-positive patients was larger than that of node-negative patients, no matter whether examined by FCM or pathology. LN metastases were more prominent in large-sized tumors.

## 4 Discussion

In this study, we employed a combination of the tumor molecular markers CK20, Pan-CK, and EpCAM to detect metastatic tumor cells in LNs from (CRC) patients. Our findings demonstrated that the utilization of tFCM allowed for accurate identification of different levels of tumor cells in LNs, including MIC and ITC. Consequently, FCM holds the potential to facilitate rapid nodal staging by assisting surgeons.

It typically takes several days for their identification through the examination of MAC-LN sections under a microscope. Furthermore, if MIC and ITC cannot be observed or diagnosed via H&E staining, additional IHC testing is required, which can extend the diagnosis time to at least a week. In contrast, the primary advantage of FCM lies in its reagent cost parity with traditional pathological analysis while significantly economizing time. A single staff member can complete the entire LN FCM assessment within 4 h, whereas pathological examinations necessitate at least 2–4 days and can only detect metastases in fewer than 1/50 LNs. This method significantly reduces the time required for diagnosis and enhances the accuracy of detection, particularly in cases of MICs occurring within the tumor. Furthermore, with further optimization, it can offer valuable assistance to surgeons during procedures.

Previous studies have indicated that patients with cytokeratin-positive cells in LNs, detected by IHC, exhibit a poor prognosis [20]. Cytokeratins (CK) are intracellular proteins that can serve as applicable markers for metastasis detection using FCM. However, since cells must undergo fixation and embedding before FCM analysis, the number of false positive events may increase, as demonstrated by positive events in PBMCs when using CK20 alone. Moreover, the accuracy and sensitivity of using two cytokeratins in combination with the cell surface membrane protein EpCAM were higher than those achieved by utilizing either cytokeratin alone or both cytokeratins simultaneously. Previous research has focused on using one or two tumor markers to develop FCM, but the accuracy of FCM in detecting MIC or ITC remains uncertain [21]. Therefore, in our study, the background fluorescence of the node group was deduced using an isotype control group. It is worth noting that isotype control is typically used as gating in FCM to define true positive and negative events, rather than to calculate positive events, and no isotype can perfectly match the specific antibody used [22,23]. Considering an LN size of approximately 6 × 5 × 4 mm, half of it can yield around 50 sections of 4 μm thickness. However, in routine pathology, only two–five sections are typically examined, resulting in a significant proportion (90%) of the LN going unexamined and thereby posing a high risk of false negatives. A MIC measuring 0.2 mm accounts for merely 0.16% of the total LN cell count, while ITC corresponds to a mere 0.032% of the total LN cell count. In our spiking experiment utilizing the three tumor markers with FCM, we were able to detect 0.00123% of SW480 cells from PBMCs, indicating that the method is stable and sensitive enough for ITC detection. Furthermore, no positive events for CK20-EpCAM-PanCK were detected in PBMCs, LNs without tumor cells, or several pathologically negative nodes. To a certain extent, this substantiates the potential to minimize false positive occurrences when employing the tripartite tumor marker approach. Prior research has indicated that over 95% of CRC cases are classified as adenocarcinomas [24]. Rare histological types encompass six varieties, including squamous cell carcinoma, neuroendocrine tumor, soft tissue sarcoma, gastrointestinal stromal tumor, non-Hodgkin lymphoma, and melanoma. In this study, the FCM antibodies primarily target epithelial tissue origins, which may lead to potential oversight of other rare CRC histological types. Caution must be exercised in their application. Within our study, one case of neuroendocrine carcinoma exhibited negative results in both pathological and flow cytometric examinations of three LNs. Consequently, the possibility of LN metastasis in this patient cannot be entirely ruled out. On another note, multi-parameter FCM presently has the capability to simultaneously assess more than ten antibodies. Hence, there is promise for further optimization of flow cytometric testing protocols. This optimization aims not only to detect tumor metastases but also to scrutinize the immunophenotype of immune cells within LNs, thereby offering additional insights into the prognostic characteristics of CRC [25]. The Meta-Analysis led by Mao et al. has delineated several pivotal risk factors defining LN metastasis in patients afflicted with thyroid carcinoma. These encompass age (<45 years), gender (male), multifocality, tumor dimensions (>1 cm), tumor location (upper one-third), capsular infiltration, and thyroid extracapsular extension [26]. Tsuchihashi and colleagues have established a model for forecasting the risk factors associated with LN metastasis in submucosal CRC (SM CRC). They partition patients based on the presence or absence of LN metastasis in SM CRC into three risk factors: lymphatic vessel invasion, budding grade, and depth of submucosal infiltration [27]. The foundation of such investigations into prognostic factors for LN metastasis hinges on the precise identification of LN metastasis in each case. Herein, tFCM offers a method that combines various immune markers not only for detecting metastasis but also for assessing the invasive potential of draining tumor cells in successive LNs, offering distinct advantages over traditional pathological examinations. Additionally, our future focus will be on optimizing the FCM procedure to reduce the time required and to explore the functional mechanisms underlying metastatic tumor cells in LNs.

## 5 Conclusion

tFCM represents a rapid, accurate, and automated objective approach for detecting LN metastases in CRC patients. It holds promise for nodal staging and presents a potential alternative to subjective microscopic pathological sections in the diagnostic process for LN involvement.
